# “The Perfect Swarm” – Flooding across Northern Victoria leads to intensified mosquito breeding and subsequent re-emergence and transmission of Murray Valley encephalitis virus during the 2022-23 mosquito season

**DOI:** 10.1371/journal.pntd.0013407

**Published:** 2025-08-18

**Authors:** Peter J. Neville, Rachel N. Evans, Helen O’Brien, Peter T. Mee, Natasha D. Brohier, Nicole Hughes

**Affiliations:** 1 Communicable Disease Prevention and Control, Department of Health, Melbourne, Victoria, Australia; 2 Agriculture Victoria Research, AgriBio Centre for AgriBioscience, Bundoora, Melbourne, Victoria, Australia; University of Cincinnati, UNITED STATES OF AMERICA

## Abstract

**Background:**

The sporadic yet explosive nature of Murray Valley encephalitis virus (MVEV) in the southeast of Australia has led to several hypotheses to explain the viruses temporal and spatial patterns in the region. These theories, relying on the presence of flooding events, include the role of migratory waterbirds as carriers of MVEV from endemic areas in northern Australia or the re-appearance of MVEV from isolated, cryptic habitats. Here we describe the environmental, climatic and entomological factors that led to the development of the “perfect swarm” and re-emergence of MVEV in Victoria during the 2022–23 mosquito season, allowing greater scrutiny of the proposed theories for MVEV outbreaks in the southeast of Australia.

**Methodology:**

Mosquitoes were collected using Encephalitis Virus Surveillance Carbon Dioxide (EVS CO_2_) traps that were set weekly across 17 northern Victorian Local Government Authorities (LGAs). Trapped mosquitoes were counted, speciated and screened for known viruses of public health significance. Mosquito abundance and species composition was compared with climatic variables including temperature and rainfall.

**Results:**

A positive Southern Oscillation Index and a significant rainfall event in October 2022 led to flooding in central and northern Victoria that persisted for many months. Dominance of the mosquito fauna by *Culex australicus* in October to December 2022, followed by *Culex annulirostris* over January to March 2023 provided the vectors for amplification and subsequent transmission of MVEV within hosts, leading to spill-over into human populations for the first time in Victoria in almost 50 years. Mosquito surveillance systems detected MVEV in 48 mosquito traps over a 13-week period commencing in the first week of 2023. Virus positive species-specific pools included *Cx. australicus* and *Cx. annulirostris*, implicating both species as playing a role in MVEV amplification and/or transmission. Typing of MVEV was determined to be closely related to MVEV serotype G1A sub-lineage, which had previously only been detected in northwest Australia and from a human case in the Northern Territory.

**Conclusions:**

The detection of MVEV during the 2022–23 mosquito season in south-eastern Australia provides greater context for understanding the re-appearance of MVEV in this region, with increased evidence implicating the role of migratory waterbirds, in response to flooding events, as carriers of MVEV into south-eastern Australia.

## Author summary

Murray Valley encephalitis virus (MVEV) is a rare but potentially fatal disease that is spread to humans through the bite of an infected mosquito. The symptoms can progress quickly from fever, headaches and neurological dysfunction to neuropathy, coma, and death. Within Australia, MVEV is considered a condition of urgent public health priority, with most State Health Departments managing mosquito surveillance programs to provide an early warning system to detect Murray Valley encephalitis (MVE) and other mosquito-borne viruses of public health significance and deploy integrated mosquito management principles to control mosquito populations and protect public health.

This study documents the impact of the environmental and climatic conditions that led to the first outbreak of MVEV disease in Victoria in 50 years. Sudden and significant rainfall along the Murray River, led to flooding along the Victorian/New South Wales border aligning with the development of mosquito populations in the peak summer months creating the “perfect swarm” for disease amplification, transmission and ‘spill-over’ into human populations. Confirmation of the G1A MVEV lineage, previously recorded in northwest Australia, although sharing a common ancestry with early Victorian isolates, suggests migratory waterbirds as the vehicle for movement of MVEV from endemic to non-endemic regions of Australia.

## Introduction

Murray Valley encephalitis virus (MVEV) is a mosquito-borne flavivirus and a member of the Japanese encephalitis serogroup that includes Japanese encephalitis virus (JEV), West Nile virus (WNV) Kunjin virus (KUNV) subtype and other encephalitides from around the world [1]. In Australia, Murray Valley encephalitis (MVE) is endemic in northern Australia, with rare outbreaks occurring in the southeast of the country, under specific environmental and climatic conditions. The majority of MVEV infections are asymptomatic and subsequently undiagnosed and unreported to health authorities, although it is estimated that 1:150–1:1,000 infections lead to clinical encephalitis [2,3]. Symptoms of MVE usually develop after an incubation period of up to four weeks and include the development of fever, headaches, altered mental state and malaise. These symptoms can progress to further neurological deterioration, including tremors, cranial nerve palsies, peripheral neuropathy, coma and death [1]. In children, the signs and symptoms of MVE can be difficult to ascertain, but often result in tremors and convulsions, floppiness, and an unresponsive state. Historically, death is reported in 15–30% of reported cases, with 30–50% of patients recovering with neurological sequelae, while only 40% recover completely [4]. However, during the 2022–23 MVEV outbreak in Victoria, a 70% fatality rate was reported from notified cases [5]. MVEV is a nationally notifiable disease in Australia and a condition of public health importance for Victoria.

MVEV is endemic in northern Australia [6], with enzootic cycles predominantly between waterbird and mosquito (primarily *Culex annulirostris*) populations with regular spill-over into human populations at low frequencies [1]. In south-eastern Australia, MVEV outbreaks are rare and irregular, likely responsible for reported encephalitic outbreaks during the 1917–18, 1922 and 1925 mosquito seasons [1,7] that were attributed to “Australian X” disease. However, it was not until the 1950–51 outbreak that MVEV was first identified as the likely causative pathogen [8,9]. Further sporadic cases of MVE were reported in south-eastern Australia in 1956 and 1971 [7] before the most significant outbreak in 1974 with 58 confirmed cases, of which 22 were recorded in Victoria [2,4,10].

Mosquito surveillance and control activities in southeastern Australia were mostly informal and ad hoc prior to the 1974 outbreak. The large number of deaths and associated impacts on local communities resulted in the establishment of a more coordinated mosquito surveillance and control program, with seasonal weekly adult mosquito trapping becoming commonplace. However, this only informed the abundance and species composition of mosquitoes associated with flaviviruses, as the cell culture techniques were restricted to the detection of alphaviruses. Concurrently, flavivirus detection was obtained by a parallel surveillance system that involved collection of blood samples from strategically positioned flocks of sentinel chickens that acted as a proxy for wild waterbirds, the natural zoonotic reservoir.

These surveillance activities continued for several decades, with modifications mostly dictated by funding levels or as a result of local government boundary changes. By the time of the next detection of MVE that included south-eastern Australia (with two human cases reported in both New South Wales and South Australia) during the 2010–11 season, some local governments were still undertaking surveillance activities that were largely unchanged for nearly 40 years, yet others had long retired their field programs and redirected resources to other priorities, leaving surveillance gaps in areas of concern.

The 2010–11 MVE detections were precipitated by a multi-year series of high-rainfall weather events across the southeast of Australia, including substantial 2010 spring and 2011 summer floods that led to intense mosquito proliferation [4]. Seventeen human cases were recorded nationally, four in south-eastern Australia (two in New South Wales and two in South Australia) [4]. Sentinel chicken seroconversions were reported for south-eastern states with 90 MVEV seroconversions in Victoria and 24 in New South Wales [4]. MVEV was also detected in central nervous system tissue from five horses in Victoria [11]. Despite widespread detection of MVEV in south-eastern Australia in 2011, a serosurvey of blood donors and stored serum specimens in the Murray River region of Victoria found little recent exposure (<5%) to MVEV [12].

Over the preceding years, little information emerged to help explain these observations, but in response to the event, Victoria made modifications to its surveillance program. A review of previous outbreaks indicated that the long-standing sentinel chicken surveillance program in Victoria was ineffective as an early warning system, as human cases of disease were notified before seroconversions in chickens were confirmed. Advances in laboratory molecular assays and the implementation of bulk mosquito trap processing methods allowed for the retirement of this vertebrate flavivirus surveillance program in October 2020.

During the 2022–23 mosquito season, the re-emergence of MVEV was detected across south-eastern Australia. In Victoria, this represented the largest outbreak of MVE since 1974 with a total of six confirmed human cases of MVE reported, of which five subsequently died from the disease [5]. There were also 53 detections of MVEV from mosquito pools across 48 mosquito traps located across northern and north central Victoria.

While climate (particularly rainfall) has been implicated in the emergence of MVEV in southern Australia and predictive models have been developed (the Forbes [13] and Nicholls [14] hypotheses), zoonotic, vector-borne viruses are complex and there are several other factors that influence the development of an outbreak. Two theories have long been proposed to explain the re-emergence of MVEV in the southeast of Australia over recent decades. The first, that MVEV exists at low levels in isolated cryptic natural environments, circulating between mosquitoes and waterbird populations, leading to outbreaks under significant rainfall and flooding conditions, when mosquito and bird populations increase and disperse more widely into surrounding flooded habitats [15]. The second hypothesis suggests that MVEV is re-introduced into south-eastern Australia by migratory waterbirds from northern Australia (where MVEV is enzootic) after significant rainfall and flooding in the southeast [7,16]. Upon arrival in flooded wetlands, mosquitoes enhance amplification of the virus by infecting local bird populations, leading to an increase in viral load within the environment and potential transmission (‘spill-over’) to human populations.

A third theory, for re-emergence of MVEV in northern Australia, implicates vertical transmission of virus in desiccant resistant mosquito eggs, providing a source of virus in subsequent years. Studies in the Kimberley region of Western Australia have demonstrated reactivation of MVEV through vertical transmission in *Aedes tremulus* eggs [17]. Thus, emerging mosquitoes in subsequent seasons may already be infected with MVEV.

There are four different genotypes of MVEV, which are named G1 to G4. Currently, the genotypes G1 and G2 are circulating in Australia, while G3 and G4 have not been detected in recent years. It is believed that the latter two genotypes are either confined to Papua New Guinea or are no longer in circulation [18]. Within the G1 genotype, there are two distinct sublineages: G1A, which is found only in the northwest of Australia, and G1B, which is found across Australia [18].

Frequent circulation of MVEV in northern Australia based on molecular evidence indicates virus emergence from a constrained enzootic focus [19–22]. However, in the southeast, MVEV activity can be absent for extended periods, before sporadically re-emerging, leading to significant public health impacts [5].

Considering the significant re-emergence of MVEV in Victoria during the 2022–23 mosquito season, we explore the entomological component of the Victorian Arbovirus Disease Control Program (VADCP) and the environmental and climatic factors that intensified mosquito development and led to the amplification and subsequent transmission of MVEV. This disease outbreak provided the opportunity to consider the previously suggested theories for MVEV re-emergence in Victoria against the surveillance data collected during the 2022–23 mosquito season.

## Methods

### Mosquito surveillance

The VADCP is a state-wide collaborative program coordinated by the Victorian Department of Health and delivered by participating local councils within regions of the state considered to be high-risk for mosquito-borne viruses. The aims of the program are to provide an early warning surveillance system for mosquito-borne diseases and provide intelligence to inform an active integrated mosquito management program to reduce the incidence of disease. The program conducts surveillance through weekly mosquito trapping, identification and numeration of mosquito species and testing for viruses of public health concern. In response to the information collected, an active integrated mosquito management program including physical and chemical control is implemented to reduce vector populations. Targeted public health communications are distributed to support human behaviour modification and prevention of disease.

**Fig 1:**
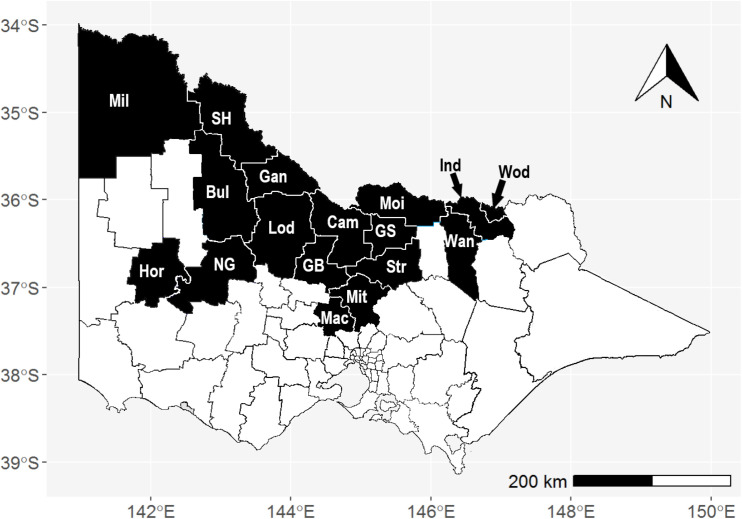
Map of Victoria, Australia, showing the 17 local government authorities that participated in adult mosquito surveillance for MVEV as part of the VADCP for the 2022–23 mosquito season. Councils shaded in black with white borders indicate councils involved in mosquito surveillance for MVEV and JEV across north central and northern Victoria (including Mil – Mildura Rural City Council, SH – Swan Hill Rural City Council, Gan – Gannawarra Shire Council, Bul – Buloke Shire Council, NG – Northern Grampians Shire Council, Hor – Horsham Rural City Council, Lod – Loddon Shire Council, Cam – Campaspe Shire Council, GB – City of Greater Bendigo, Mac – Macedon Ranges Shire Council, Mit – Mitchell Shire Council, Str – Strathbogie Shire Council, GS – Greater Shepparton City Council, Moi – Moira Shire Council, Ind – Indigo Shire Council, Wod – City of Wodonga and Wan – Rural City of Wangaratta). Base maps are derived from the Australian Bureau of Statistics postal areas (POA) shapefile POA_2016_AUST, available here. The file has been modified to include Local Government Authority boundaries and is distributed under the Creative Commons Attribution 4.0 International, Accessed 21/03/2025.

During the 2022–23 mosquito season, surveillance was undertaken by 23 local government authorities (LGAs) across Victoria, primarily located along the northern state border with New South Wales and extending down through central northern Victoria (17 LGAs), as well as some metropolitan (3 LGAs) and coastal regions of the state (4 LGAs) (Fig 1). Only 17 LGAs are included in this analysis, as these areas are at risk from MVEV based on past disease outbreaks and surveillance data.

Encephalitis Virus Surveillance carbon dioxide (EVS CO_2_) light traps baited with dry ice pellets were set on a weekly basis from September 2022 to mid-June 2023. Each council set a minimum of four traps per week, at long-term surveillance sites with previous detections of arboviruses of public health significance, between wetland habitats and key population centres. Additional surveillance traps were set by LGAs to investigate public complaints, monitor mosquito populations near outdoor public events, or at recreation reserves where the public congregate. Traps were hung on the western side of sturdy vegetation 1.5m from the ground during the afternoon and collected the following morning. Mosquitoes collected overnight were killed by placing in a freezer for an hour before being transferred into labelled petri dishes and transported by overnight mail to Agriculture Victoria Research (AVR) in cooler bags with ice bricks to maintain the cold chain.

Upon arrival at the laboratory, samples were unpacked and placed into -20-degree Celsius freezers to maintain the cold chain while awaiting processing. Samples were placed onto cold tables for morphological identification using a taxonomic key [23]. Samples containing less than 150 mosquitoes were fully identified to genus and species, with subsampling of species composition occurring for samples containing greater than 150 mosquitoes (weight extrapolated to provide species composition for the total trap weight). Species composition data was entered into a web-based data recording system, providing timely reporting, intelligence and data to LGAs and the Department of Health. Mosquito abundances were converted to average abundance per trap, per trap night, throughout the season to standardise data and allow comparison over time and space.

### Sample preparation for virus screening

After identification up to 1,000 mosquitoes per trap per trap night were placed in a 50 mL conical tube. Traps that contained more than 1,000 mosquitoes were placed into multiple tubes, containing no more than 1,000 mosquitoes per tube. A single 15mm metal bead was added to each tube and 2mL of MEM medium (8% FBS, 0.1% amphotericin, 1% [penicillin and streptomycin], 10% L-glutamine and 1% HEPES) per 100 mosquitoes. Mosquitoes were homogenised using a 2010 Geno/Grinder (Thomas Scientific) at two cycles of 1,000 strokes/minute for 1.5 minutes. Samples were then centrifuged at 2,000 RPM for 20 minutes. A 50 µL aliquot was removed and used for extraction using the MagMax Viral RNA Isolation Kit (Applied Biosystems) on a KingFisher (ThermoFisher) magnetic particle processor, following manufacturer’s recommendations.

### RT-qPCR

Each mosquito extract was tested for five viral targets using reverse transcription-quantitative polymerase chain reaction (RT-qPCR). Primers and probes were obtained from previously published articles for Ross River virus (RRV) [24], Barmah Forest virus (BFV) [25], West Nile (Kunjin subtype) virus (KUNV) [26], MVEV [26] and Japanese encephalitis virus (JEV) [27]. The RRV and BFV were run as a multiplex assay, as was MVEV and KUNV, with the JEV tested as a single-plex assay. PCR master mixes were prepared using AgPath-ID One-Step RT-PCR Reagent, consisting of 12.5 µL of 2X RT-PCR Buffer, 1 µL of 25X RT-PCR Enzyme Mix, 1 µL of each primer probe mix per target, 1 µL of VetMax Xeno Internal Positive Control DNA (ThermoFisher), 1 µL of VetMax Xeno LIZ assay mix (ThermoFisher), 5 µL of template, with the reaction made up to 25 µL with nuclease-free water. The RT-qPCR reactions were carried out on a QuantStudio 5 Instrument (Applied Biosystems) using the following reaction conditions, 1 cycle at 48˚C for 15 minutes, 1 cycle at 95˚C for 10 minutes, and then 45 cycles at 95˚C for 15 secs and 60˚C for 45 secs. All reactions included a no template control and a high and low positive control. A sample was determined to be positive if the VetMax Xeno assay had a cycle threshold (Ct) value between 27–30 and the target assay had a Ct value of < 37, both positive controls were detected, and nothing was detected in the negative control. If a sample tested positive, it was re-extracted and tested in duplicates to confirm the detection. Of the mosquitoes that were screened in species-specific pools, the maximum likelihood estimate (MLE) per 1,000 mosquitoes tested (bias-corrected MLE for point estimation of infection rate and a skew-corrected score CI) was calculated from the pooled samples.

Positive detections of MVEV were confirmed by following a metatranscriptomic approach. Nucleic acid was extracted using a PureLink Viral RNA/DNA Mini Kit (Invitrogen) and quantified using a Qubit 1xdsDNA High Sensitivity Assay Kit on a Qubit Fluorometer (Invitrogen). Sequencing libraries were prepared using a Universal Plus Total RNA-Seq library preparation kit with NuQuant (Tecan) with custom mosquito rRNA depletion probes as previously outlined [28]. Libraries were quantified with a HsD1000 ScreenTape on a 2200 TapeStation (Agilent Technologies) and a Qubit 1x dsDNA High Sensitivity Assay Kit on a Qubit Fluorometer (Invitrogen). Libraries were pooled in equimolar ratios and sequenced on an Illumina NovaSeq 6000, at a depth of 6 GB per sample. Sequences were mapped to a curated database containing Australian arbovirus genomes as previously outlined [28] containing 80 arboviruses, representing nine viral families based on [7] and [29]. Bowtie 2 v2.4.5 [30] was used to perform the mapping and the percentage of arbovirus genome coverage by reads was measured using BBMap pileup [31].

### Meteorological data

Rainfall (mm), maximum temperature (°C) and minimum temperature (°C) was accessed from the Bureau of Meteorology, Commonwealth of Australia, from six weather stations across northern Victoria within LGAs that conducted mosquito surveillance (including Mildura - Mildura Airport, Swan Hill – Swan Hill Aerodrome, Gannawarra - Kerang Model Farm, Campaspe - Echuca Aerodrome, Moira – Cobram Goulburn Murray and Wodonga – Wodonga). Meteorological data for the past 25 years was converted to average monthly and average weekly values over the study period for comparison with average monthly/weekly mosquito abundance for corresponding trap collections.

## Results

**Fig 2:**
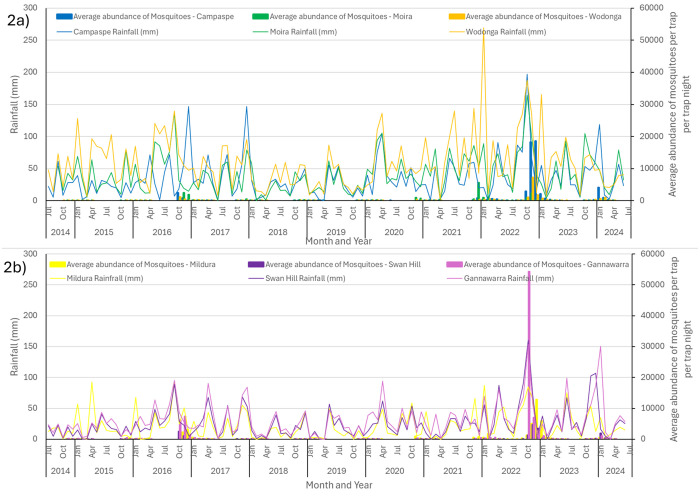
Monthly rainfall (mm) recorded at six weather stations along the Murray River from July 2014 to June 2024 and the corresponding abundance of mosquitoes per trap per trap night each month. Fig 2A) Eastern headwaters of the Murray River including Wodonga – Wodonga, Moira – Cobram Goulburn Murray, and Campaspe - Echuca Aerodrome weather stations; Fig 2B) western section of the Murray River including Gannawarra - Kerang Model Farm, Swan Hill – Swan Hill Aerodrome and Mildura - Mildura Airport weather stations. Meteorological data collected from the Bureau of Meteorology 2024, Government of Australia.

Rainfall patterns over the past decade indicate that the eastern headwaters of the Murray River receive slightly higher and more regular rainfall than LGAs along the western end of the river system (Fig 2; Table 1). The LGAs of Wodonga, Moira and Campaspe in the east, saw rainfall over 100mm in at least one month, during seven out of ten years for Wodonga, 5 years for Campaspe and 4 years for Moira. In contrast, rainfall over 100mm only occurred in any month in two out of ten years for Gannawarra and Swan Hill and never reached 100mm in any month for Mildura further along the river system (Fig 2).

**Table 1:** A comparison of long-term mean monthly and annual rainfall (mm) collected over 25 years with the mean average monthly and annual rainfall (mm) recorded in 2022 alone, across six weather stations from the headwaters of the Murray River in the east to the mouth of the Murray River system in the west including Wodonga, Moira, Campaspe, Gannawarra, Swan Hill and Mildura. Meteorological data collected from the Bureau of Meteorology 2024, Government of Australia.

Local Government Authorities and associated weather station from east (headwaters) to west (mouth) along the Murray River	Long-term mean monthly rainfall (mm) and long-term mean annual rainfall (mm)												
	1997–2021												
	Jul	Aug	Sep	Oct	Nov	Dec	Jan	Feb	Mar	Apr	May	Jun	Annual
Wodonga - Wodonga	80.1	76.7	62.4	67.9	51.4	48.3	73.3	27.3	54.5	51.1	62.3	73.9	60.8
Moira - Cobram Goulburn Murray	41.6	43.9	42.3	42.7	42.4	37.6	42.4	43.9	47.9	35.0	34.9	38.7	41.1
Campaspe - Echuca Aerodrome	40.5	42.0	38.4	42.7	33.3	29.9	30.0	20.8	23.9	29.7	32.2	38.7	33.5
Gannawarra - Kerang Model Farm	34.5	35.0	34.5	31.3	35.6	30.5	33.0	21.8	24.8	30.3	28.5	34.0	31.2
Swan Hill - Swan Hill Aerodrome	25.6	26.7	27.5	27.3	44.1	24.8	25.2	19.0	16.5	23.7	26.0	24.5	25.9
Mildura - Mildura Airport	24.5	25.1	26.3	29.3	27.2	24.2	22.2	22.7	18.3	17.8	17.8	18.7	22.8
													
**Local Government Authorities and associated weather station from east (headwaters) to west (mouth) along the Murray River**	**Mean monthly rainfall (mm) for July 2022 to June 2023 and corresponding mean annual rainfall (mm)**												
	**2022**						**2023**						**2022–23**
	**Jul**	**Aug**	**Sep**	**Oct**	**Nov**	**Dec**	**Jan**	**Feb**	**Mar**	**Apr**	**May**	**Jun**	**Annual**
Wodonga - Wodonga	19.6	####	134.4	187.8	134.6	11.7	165.6	7.4	67.4	76.7	53.6	99.0	89.3
Moira - Cobram Goulburn Murray	13.0	75.7	84.3	164.2	98.8	39.8	25.8	4.3	18.7	62.4	18.0	93.0	58.2
Campaspe - Echuca Aerodrome	18.8	86.5	75.4	196.8	66.0	17.4	55.0	7.1	32.4	47.8	23.6	91.3	59.8
Gannawarra - Kerang Model Farm	16.7	48.1	68.0	153.6	57.8	4.0	25.0	5.8	21.6	47.8	12.8	99.2	46.7
Swan Hill - Swan Hill Aerodrome	9.4	52.2	90.6	160.6	73.0	4.8	37.6	0.2	19.2	39.4	12.4	67.2	47.2
Mildura - Mildura Airport	3.8	41.4	55.9	85.1	65.2	8.0	19.7	0.0	7.3	22.7	12.1	75.0	33.0

**Fig 3:**
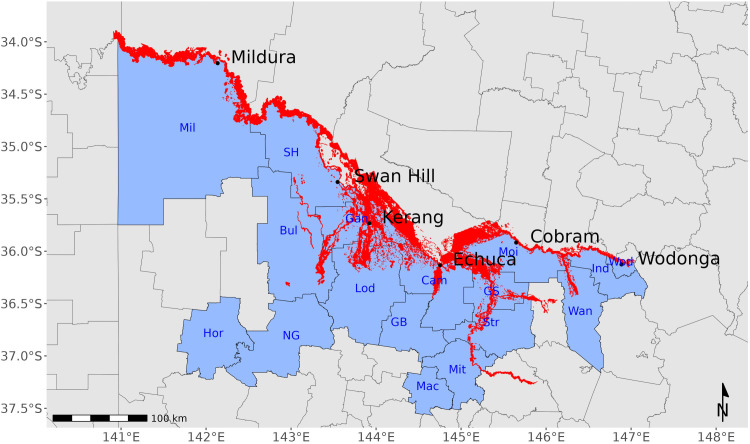
Satellite imagery of the derived flood extent (red) generated by automated and manual processing of data at a given point in time for October 2022 (may not represent the maximum flood extent). Emergency Management Victoria, State Government Victoria. The file has been modified to include Local Government Authority boundaries and is distributed under the Creative Commons Attribution 4.0 International, Accessed 21/03/2025. Available at: Victorian Flood History October 2022 Event Public - Dataset - Victorian Government Data Directory.

Three consecutive La Niña weather systems across Eastern Australia between 2020 and 2023 and associated rainfall during the last quarter of the 2021–22 season led to waterlogged soils over much of central and northern Victoria. Further heavy rainfall recorded for Swan Hill and Echuca in April 2022 and Wodonga in early June 2022 (Fig 2) maintained saturated soil moisture content. The following wet spring culminated in significant rainfall between the 12–14^th^ October 2022, leading to flooding along the Murray River between New South Wales and Victoria. Rainfall was double the long-term monthly average for all six LGAs along the Murray River system in September 2022 and increased to be almost three times the average rainfall in October 2022 for Wodonga and Mildura, 3.9 times the average for Moira and over 4 times the monthly October rainfall in Campaspe, Gannawarra and Swan Hill (Table 1). This accumulation of water within the Murray River system ensured flood waters downstream continued to overflow the riverbanks in the northwest of Victoria and into South Australia, where the river system empties into the Southern Ocean (Fig 3).

Long-term historical trends in rainfall also indicate a surge in the average number of mosquitoes collected per trap, per trap night in the months following significant rainfall events of 100mm or more at multiple locations along the Murray River. The average monthly mosquito catches per trap, per trap night are seen to increase in October 2016, November 2021, October 2022 and again in January 2024 (Fig 2). These dates are related to significant rainfall of 100mm or more in at least two locations simultaneously along the Murray River just prior to the corresponding increase in mosquito populations.

In general, average maximum temperatures (^0^C) recorded in 2022–23 were consistently below the long-term monthly averages for all locations along the Murray River (Table 2). The variance between the long-term average maximum temperature and the maximum temperatures recorded in winter of 2022 and 2023 was generally less than half a degree across each of the sites examined but increased to two degrees during peak summer periods. All six locations examined showed maximum temperatures to be at least 0.5 degrees below the long-term maximum temperatures in September, October, November and December 2022 and in April 2023. The annual difference in maximum temperatures for all locations examined showed a slightly lower maximum temperature in 2022–23, compared to the long-term average maximum temperature for the same locations across Victoria, varying by approximately 1.0 to 1.5 degrees Celsius.

**Table 2:** A comparison of long-term average monthly and annual maximum temperatures (^0^C) collected over 28 years with the mean average monthly and annual maximum temperatures (^0^C) recorded in 2022 alone, across six weather stations from the headwaters of the Murray River in the east to the mouth of the Murray River system in the west including Wodonga, Moira, Campaspe, Gannawarra, Swan Hill and Mildura. Meteorological data collected from the Bureau of Meteorology 2024, Government of Australia.

Local Government Authorities and associated weather station from east (headwaters) to west (mouth) along the Murray River	Long-term mean monthly maximum temperature (^0^C) and long-term mean annual maximum temperature (^0^C)												
	1997–2021												
	Jul	Aug	Sep	Oct	Nov	Dec	Jan	Feb	Mar	Apr	May	Jun	Annual
Wodonga - Wodonga	13.4	15.0	18.4	22.1	26.6	29.8	32.7	31.4	28.1	22.8	17.6	14.1	22.6
Moira - Cobram Goulburn Murray	13.7	15.3	18.9	23.0	27.6	30.5	33.1	31.8	28.3	23.2	18.1	14.5	23.2
Campaspe - Echuca Aerodrome	14.1	15.8	19.3	23.1	27.1	29.9	32.4	31.5	28.0	23.0	18.1	14.7	23.1
Gannawarra - Kerang Model Farm	14.7	16.6	20.2	23.9	27.8	30.5	32.9	32.0	28.4	23.6	18.5	15.1	23.7
Swan Hill - Swan Hill Aerodrome	14.8	16.8	20.4	24.3	28.1	30.8	33.3	32.3	28.7	23.7	18.8	15.3	23.9
Mildura - Mildura Airport	16.0	18.0	21.8	25.2	28.7	31.4	33.8	32.7	29.0	24.3	19.5	16.3	24.7
													
**Local Government Authorities and associated weather station from east (headwaters) to west (mouth) along the Murray River**	**Mean monthly maximum temperature (** ^**0**^ **C) for July 2022 to June 2023 and corresponding mean annual maximum temperature (** ^**0**^ **C)**												
	**2022**						**2023**						**2022–2023**
	**Jul**	**Aug**	**Sep**	**Oct**	**Nov**	**Dec**	**Jan**	**Feb**	**Mar**	**Apr**	**May**	**Jun**	**Annual**
Wodonga - Wodonga	13.9	15.4	17.4	20.7	22.1	27.4	31.0	29.6	27.6	21.6	15.8	14.1	21.4
Moira - Cobram Goulburn Murray	14.1	15.5	17.0	20.3	22.7	28.7	32.4	31.5	28.2	21.9	16.6	14.0	21.9
Campaspe - Echuca Aerodrome	14.2	15.6	16.8	20.6	22.8	28.3	31.6	30.4	26.7	22.0	17.0	14.6	21.7
Gannawarra - Kerang Model Farm	14.4	16.5	17.6	20.7	22.8	28.8	32.4	31.1	27.6	22.0	17.5	15.3	22.2
Swan Hill - Swan Hill Aerodrome	14.6	16.5	17.7	21.0	23.2	29.1	32.7	31.4	28.3	22.3	18.1	15.5	22.5
Mildura - Mildura Airport	15.4	18.2	19.2	22.5	24.1	30.1	34.0	32.7	29.0	23.3	19.3	16.8	23.7

Mean minimum temperatures in 2022–23 were slightly higher (by about half a degree to a degree Celsius) than the long-term average for the equivalent weather stations along the Murray River (Table 3) for most locations in August, September and October 2022 and in June 2023. However, the variance was inconsistent throughout the year, with the months of July, November, and December 2022, February and May 2023 being up to two degrees cooler compared to the long-term average. However, on an annual basis, the average minimum temperatures in 2022 were 0.2-to 1.0-degree warmer than the long-term mean minimum temperatures across all locations.

**Table 3:** A comparison of long-term average monthly and annual minimum temperatures (^0^C) collected over 28 years with the mean average monthly and annual minimum temperatures (^0^C) recorded in 2022 alone, across six weather stations from the headwaters of the Murray River in the east to the mouth of the Murray River system in the west including Wodonga, Moira, Campaspe, Gannawarra, Swan Hill and Mildura. Meteorological data collected from the Bureau of Meteorology 2024, Government of Australia.

Local Government Authorities and associated weather station from east (headwaters) to west (mouth) along the Murray River	Long-term mean monthly minimum temperature (^0^C) and long-term mean annual minimum temperature (^0^C)												
	1997–2021												
	Jul	Aug	Sep	Oct	Nov	Dec	Jan	Feb	Mar	Apr	May	Jun	Annual
Wodonga - Wodonga	3.1	3.7	5.8	8.3	12.0	14.4	16.9	16.5	13.2	8.7	5.4	3.6	9.3
Moira - Cobram Goulburn Murray	3.4	3.6	5.1	7.5	11.4	13.6	16.1	15.8	13.0	9.2	6.1	4.0	9.1
Campaspe - Echuca Aerodrome	3.4	4.0	5.7	8.0	11.3	13.4	15.6	15.5	12.7	9.1	6.1	4.1	9.1
Gannawarra - Kerang Model Farm	4.0	4.7	6.7	9.0	12.2	14.2	16.3	16.1	13.3	10.0	6.9	4.7	9.9
Swan Hill - Swan Hill Aerodrome	3.6	4.1	5.8	8.1	11.8	13.9	16.2	15.9	12.9	9.1	6.4	4.3	9.3
Mildura - Mildura Airport	4.4	5.2	7.6	10.1	13.4	15.5	17.6	17.1	13.9	10.1	7.2	5.0	10.6
													
**Local Government Authorities and associated weather station from east (headwaters) to west (mouth) along the Murray River**	**Mean monthly minimum temperature (** ^**0**^ **C) for July 2022 to June 2023 and corresponding mean annual minimum temperature (** ^**0**^ **C)**												
	**2022**						**2023**						**2022–23**
	**Jul**	**Aug**	**Sep**	**Oct**	**Nov**	**Dec**	**Jan**	**Feb**	**Mar**	**Apr**	**May**	**Jun**	**Annual**
Wodonga - Wodonga	1.2	5.7	6.7	10.4	10.7	12.8	16.6	14.8	14.0	8.7	4.8	5.2	9.3
Moira - Cobram Goulburn Murray	1.0	4.5	5.6	9.4	10.1	12.3	16.1	14.3	13.5	9.3	5.2	5.8	8.9
Campaspe - Echuca Aerodrome	2.2	5.8	7.5	10.8	10.2	12.3	15.6	13.8	13.1	9.4	5.0	5.9	9.3
Gannawarra - Kerang Model Farm	3.1	5.5	7.6	10.5	11.1	13.1	15.7	14.8	13.4	10.1	6.0	6.3	9.8
Swan Hill - Swan Hill Aerodrome	2.8	4.9	6.4	9.9	10.0	12.6	15.7	14.4	13.0	9.6	5.3	6.5	9.3
Mildura - Mildura Airport	3.6	6.1	7.6	11.5	10.9	14.4	17.0	15.2	13.3	9.8	5.8	7.5	10.2

During the 2022–23 mosquito season in north central and northern regions of Victoria, a total of 1,211 EVS/CO_2_ light traps were set by 17 LGAs (including Mildura Rural City Council, Swan Hill Rural City Council, Gannawarra Shire Council, Buloke Shire Council, Northern Grampians Shire Council, Horsham Rural City Council, Loddon Shire Council, Campaspe Shire Council, City of Greater Bendigo, Macedon Ranges Shire Council, Mitchell Shire Council, Strathbogie Shire Council, Greater Shepparton City Council, Moira Shire Council, Indigo Shire Council, City of Wodonga and the Rural City of Wangaratta). This included traps set at a total of 237 unique locations and generated a total of 1,027,867 mosquitoes (with 150 mosquitoes per trap identified and weight extrapolated to provide species composition for the total trap weight) throughout the season.

**Fig 4:**
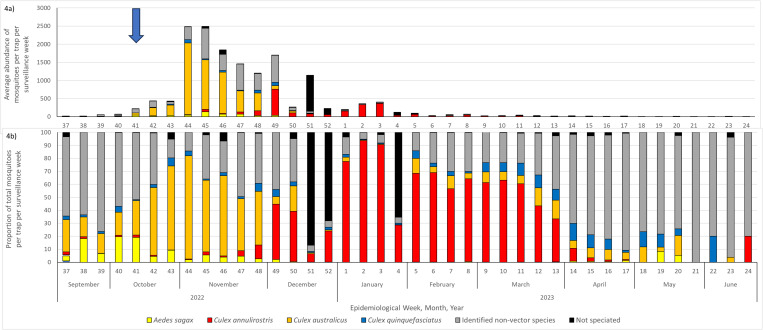
Mosquito species abundance and composition per trapping week collected from EVS CO_2_ light traps deployed across 17 north-central and northern LGAs during the 2022–23 mosquito season. Fig 4A) describes the average mosquito species abundance per trap per trap night and Fig 4B) represents the percentage composition of mosquito species per trap per trap night. Where coloured mosquito species indicate confirmed or implicated vectors of MVEV, greyed species indicate mosquitoes currently unknown to transmit MVEV and the blue arrow indicates the timing of rainfall events that led to flooding across the region.

A total of 28 mosquito species were identified from mosquito trap collections (Fig 3). Two species dominated the mosquito fauna during the 2022–23 season. *Culex australicus* (Dobrotworsky and Drummond) was present prior to the rainfall event between 12-14 October 2022 that led to flooding along the Victorian northern border, but increased from an average of 20% of adults caught per trap, per trap night to a maximum of 80% of the fauna per trap, per trap night by late October 2022 (Fig 4). *Cx. australicus* began to decline through November, being replaced by *Culex annulirostris* (Skuse) from early December 2022 through to mid-March 2023 (Fig 4). Other mosquito species had peaks in abundance that only occurred for one-to-two-week periods. The only other species exhibiting a longer-term trend was *Culex pipiens form molestus* (Forskål, 1775) (here on referred to as *Culex molestus*) that began to increase in abundance in late April 2023 and dominated the mosquito fauna until mid-June 2023, although total mosquito abundances of all species had reduced considerably by the end of the mosquito season.

A total of 2,411 mosquito traps, containing a total of 1,027,867 mosquitoes, resulted in 3,995 quantitative RT-PCR (qRT-PCR) tests were performed across Victoria during the 2022–23 season. As some traps contained more than 1,000 mosquitoes in a single trapping event, they were processed as 1,000 mosquito batches by RT-PCR. For councils considered at risk of MVEV in northern and north-central Victoria, a total of 1,211 mosquito traps comprising of 3,098 qRT-PCR tests were performed to detect flaviviruses with a total of 53 detections across 48 unique trap locations (3.96%) positive for MVEV throughout the 2022–23 extended mosquito season. Positive detections for MVEV had an average Ct value of 26.76 (range: 19.2-36.7) (S1 Table).

During the 2022–2023 season 48 traps tested positive for MVEV (out of 1,211 traps tested for flaviviruses (see Table 4)). These positive traps were collected between January 4, 2023, and March 28, 2023, spanning 29 distinct trapping locations (S1 Table). The 48 traps included 102 pools, as multiple pools were processed from some traps due to their size. Among these pools, 64.7% were mixed species pools, 18.6% were specific to *Cx. annulirostris*, 3.9% were specific to *Cx. australicus*, 3.9% were specific to *Anopheles annulipes* Walker, 3% were specific to *Cx. quinquefasciatus* Say, 2% were specific for *Aedes notoscriptus* (Skuse), 2% were specific for *Cx. molestus*, and 0.98% included species-specific pools for *Aedes vittiger* Skuse and *Aedes theobaldi* (Taylor) (Table 4). Among the positive pools, 41 were mixed species, 11 were *Cx. annulirostris* specific, and 1 was from *Cx. australicus* (Table 5). No other species-specific pools tested positive for MVEV during the 2022–23 season. The maximum likelihood estimate (MLE) of infection rate for the *Cx. annulirostris* was 1.19 (95% CI, 0.65-2.02) based on the number of MVEV positive pools per 1,000 mosquitoes tested for all *Cx. annulirostri*s tested in species specific pools over the 2022–2023 season and 3.23 (95% CI, 0.19-15.63) for *Cx. australicus (Table 5).*

**Table 4:** Breakdown of mosquito composition associated with the testing of 102 pools from the 48 MVEV positive traps collected throughout the 2022–23 mosquito season across Victoria.

Type of sample	Total number of pools from positive traps (%)	Number of pools positive for MVEV	Number of pools negative for MVEV
Mixed species pools or whole trap grinds	66 (64.7%)	41	25
*Culex annulirostris* species specific pools	19 (18.6%)	11	8
*Culex australicus* species specific pools	4 (3.9%)	1	3
*Anopheles annulipes* species specific pools	4 (3.9%)	0	4
*Culex quinquefasciatus* species specific pools	3 (2.9%)	0	3
*Aedes notoscriptus* species specific pools	2 (2%)	0	2
*Culex molestus* species specific pools	2 (2%)	0	2
*Aedes vittiger* species specific pools	1 (1%)	0	1
*Aedes theobaldi* species specific pools	1 (1%)	0	1
Total number of pools tested	102 (100%)	53	49

**Table 5:** The number of mosquitoes tested in species specific pools, the number of positive detections compared to the total number of pools tested, the maximum Likelihood Estimate (based on presence/absence of MVEV detections in species specific mosquito pools, based on uneven pool sizes) and 95% confidence Intervals for species specific pools of Cx annulirostris and Cx australicus during the 2022-23 mosquito season.

*Species*	*Number of mosquitoes*	*Number positive/number of pools tested*	*Maximum Likelihood Estimate (MLE* ^*^ ^)^	*95% Confidence Interval*
*Cx annulirostris*	*10663*	*12/218*	*1.19*	*0.65-2.02*
*Cx australicus*	*307*	*1/64*	*3.23*	*0.19-15.63*

*MLE of MVEV per 1,000 mosquitoes screened in species specific pools in Victoria.

**Fig 5:**
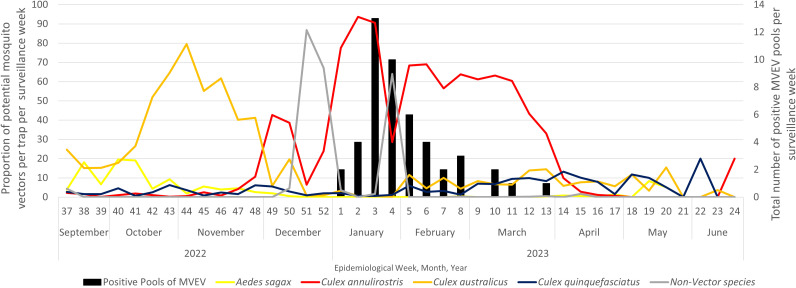
Prevalence of *Culex australicus* (orange), *Culex annulirostris* (red), *Aedes sagax* (yellow), *Culex quinquefasciatus* (dark blue), not speciated mosquitoes (grey) and the number of positive pools of MVEV (black bars) detected from mosquitoes throughout the 2022–23 season.

**Fig 6:**
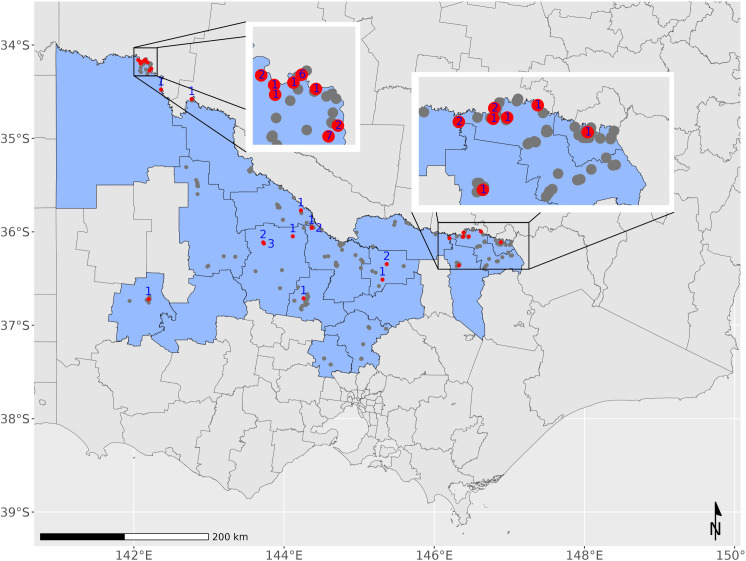
Distribution of mosquito surveillance traps across Victoria from the 17 LGAs conducting surveillance in high risk MVEV areas during the 2022–23 season - grey markers indicate traps without detection of MVEV; black circles indicate trap locations with positive detections of MVEV; red numbers indicate the number of detections at each trap site. Base maps are derived from the Australian Bureau of Statistics postal areas (POA) shapefile POA_2016_AUST, available here. The file has been modified to include Local Government Authority boundaries and mosquito trap locations distributed under the Creative Commons Attribution 4.0 International, Accessed 21/03/2025.

The first detections of MVEV in mosquitoes were in traps collected during the first week of January 2023 (epidemiological week 1). The number of MVEV detections increased throughout January before declining in February and March 2023, corresponding with the dominance of *Cx. annulirostris* during this period (Fig 5). Over the season, detections were found in traps along the Murray River from Wodonga in the east of the state through to Mildura in the northwest, having a wide geographic distribution (Fig 6).

MVEV was detected in mosquito populations in all weeks between Epi week 1 and 8 (1 January 2023 – 25 February 2023), Epi weeks 10–11 (5 March – 18 March 2023) and week 13 (26 March – 1 April 2023) (S1 Table). No detection of MVEV from trapped mosquitoes were identified during Epi week 9 (26 February to 4 March 2023) nor Epi week 12 (19 March to 25 March 2023) (S1 Table).

Mildura Rural City Council was the LGA with the highest number of detections of MVEV in mosquito populations (27 pools of mosquitoes during the 2022–23 season (S1 Table)) with MVEV consistently detected in mosquitoes over consecutive weeks (including Epi weeks 1–8 and Epi week 10). The Indigo Shire Council detected MVEV in 8 pools, the Loddon Shire Council in 6 pools, Campaspe Shire Council and Greater Shepparton City Council in 3 pools each and one pool was found positive to MVEV from each of Gannawarra Shire Council, City of Greater Bendigo, Horsham Rural City Council, Swan Hill Rural City Council, Rural City of Wangaratta, and City of Wodonga (S1 Table).

Eight mosquito pools that tested positive for MVEV underwent successful metatranscriptomic sequencing. The samples that were sequenced had an average Ct value of 24.98, and the reads were mapped against a custom database. This yielded a near-full-length (99.47%) genome with an average coverage of x128. Upon conducting a BLASTn analysis, it was identified that the virus had 99.01% genome identity to a previously sequenced MVEV isolate (MN933859) and was determined to be closely related to MVEV genotype 1 sub-lineage G1A.

## Discussion

During the 2022–23 mosquito season, Victoria experienced its largest MVEV outbreak since 1974 with six human cases and 53 mosquito viral detections of MVEV. All human cases were notified to the Department of Health subsequent to the first detections of MVEV in mosquito populations, demonstrating that mosquito arbovirus surveillance is an important tool for the early detection of mosquito-borne viruses, allowing integrated mosquito management actions to be undertaken to reduce the incidence of disease.

Three consecutive La Niña years (2020–21, 2021–22, 2022–23) had heightened concerns for the re-emergence of MVEV during the 2022–23 mosquito season, leading to a pre-season risk analysis and subsequent enhancement to mosquito surveillance prior to the onset of the season. Coupled with a positive Southern Oscillation Index, October 2022 was reported as having the highest total rainfall in northern Victoria of any month since records began in 1900 [32]. In addition, rainfall of 100mm or more, at two or more locations along the Murray River appears to trigger an increase in average mosquito populations per trap per trap night in the months post these substantial rainfall events.

Flooding events in the Murray-Darling Basin associated with the highest peaks of rainfall during 1950, 1956, 1973, 1974, 2010 were all associated with positive phases of the Southern Oscillation Index (SOI) and negative phases of the Interdecadal Pacific Oscillation (IPO) [33]. Of these years, 1950, 1973 and 1974 were all associated with outbreaks of MVE in Victoria. All MVE outbreaks in south-eastern Australia since 1917–18 can be associated with positive phases of the SOI [34] that led to heavy rainfall and/or flooding. Periods of prolonged positive SOI should be monitored in predicting future heavy rainfall and flooding events that may lead to the re-emergence of MVEV in this region.

During the 2022–23 season, rainfall was well above the long-term average for August, September, October and November 2022 leading to substantial flooding along the Murray River and associated tributaries. The flood waters were maintained through additional rainfall in January, April and June 2023, leading to a prolonged expansion to potential mosquito breeding habitats. Mean maximum daily temperatures in the 2022–23 mosquito season were consistently slightly cooler than the long-term average (by approximately one degree). However, mean minimum temperatures were elevated by approximately two degrees in August, September and October 2022, reducing variability in daily temperatures. During the 2022–23 season, flooding events were coupled with warmer minimum temperatures over much of the state [32]. These conditions are particularly suitable for enhanced mosquito development.

*Culex australicus* populations were increasing in early October 2022, dominating the mosquito fauna throughout October and November 2022. As a predominantly bird biting species [35,36], it is reasonable to assume the dominance of *Cx. australicus* could have led to an increase in amplification of virus through biting of susceptible waterbirds. Initiation and/or amplification of arbovirus activity may well be linked to population increases in *Cx. australicus* which is supported by other research [37–40]. In addition, the detection of MVEV in a single pool of *Cx. australicus* from the current study indicate they may have a role in MVEV transmission. Vector competence studies also support *Cx. australicus* as a potential vector under laboratory conditions [39,41–43].

*Cx. annulirostris* is the primary vector of MVEV in Australia [7,44] with over 90% of MVEV isolates detected from this species alone [7]. *Cx. annulirostris* dominated the mosquito fauna across north and north central Victoria from December 2022 to March 2023 with MVEV being detected from this species on 11 occasions during this period. In addition, its dominance in the environment may also indicate that many of the 41 mixed mosquito species pools could be attributed to this species as they made up 80–90% of the mosquitoes collected during this period. In laboratory studies, *Cx. annulirostris* from the Mildura region of Victoria were shown to be competent vectors of MVEV, although less effective than *Cx. annulirostris* from more northern climates [45].

MVEV has been historically detected in southern Australia, with the sub-lineage G1B being most reported, including the 2011 Victorian sentinel chicken detections [18]. However, the recent report of MVEV G1A detected in Victoria is significant because it represents the first time this sub-lineage has been found outside of northwest Australia. Previous research has suggested that G1A may have biological or ecological restrictions that keep it contained in the northwest region of Australia [18]. The detection of MVEV G1A in Victoria, although sharing a common ancestry with early Victoria isolates, provides evidence of southward movement of this sub-lineage of MVEV. This suggests that the reservoir host species had migrated south in response to significant rainfall that occurred in the southeast of Australia in 2022–23. It is worth noting that the movement of new flavivirus lineages to new areas by migrating birds has previously been associated with human outbreaks [46] and [47].

*Cx. annulirostris* is known to feed on birds and humans, establishing the transmission pathway to impact human health. Herons and egrets (Ciconiiforms) are the natural reservoirs of MVEV based on a range of studies investigating antibody prevalence, infection studies and rapid reproductive rates giving rise to susceptible cohorts [2,3,48–51]. Studies of secondary avian host have indicated that galahs (*Eolophus roseicapilla)*, sulfur-crested cockatoos (*Cacatua galerita*), corellas (*Cacatua* species), and black ducks (*Anas superciliosa*) produce a moderate viraemia to MVEV for 1–9 days and subsequently infected between 0–50% of *Cx. annulirostris* mosquitoes feeding upon them [52].

The hypothesis of re-introduction of MVEV through migration of waterbirds into the southeast of Australia is considered to be plausible based on the confirmation of MVEV sublineage G1A being detected for the first time in Victoria during the 2022–23 mosquito season [53]. Frequent circulation of MVEV based on molecular evidence indicates virus emergence from a constrained enzootic focus in northern Australia [19–22]. However, in the southeast, MVEV activity can be absent for extended periods (even decades) before re-appearing [5]. The typing of MVEV in Victorian mosquitoes to the G1A strain previously not recorded outside of northwest Australia until detected in Arnhem Land in 2018 [53] provides further evidence that the most likely pathway for G1A to enter Victoria is through the movement of host migratory waterbirds.

Studies on waterbird movements have demonstrated their adaptability in responding to changing resource availability associated with flooding events, being able to exhibit rapid spatial and temporal movements based on resource availability [54]. In addition, studies of waterbirds have demonstrated relationships between the magnitude of flooding events associated with increased breeding responses [46,47]. This is important because offspring produced during flooding events will be naïve and susceptible to infection, potentially driving the amplification of MVEV.

It is difficult to assess ornithological surveys to demonstrate movement of waterbirds in response to flooding events, as most bird surveys are opportunistic, aimed at specific research questions or difficult to design and implement in response to sudden changes in environmental conditions to be able to document patterns of movement. Host animal serosurveys (particularly for waterbirds) would provide further evidence to demonstrate the re-introduction of MVEV into the southeast of Australia via migratory waterbird movements and should be considered in future.

In comparison, the theory of localised cryptic epizootic MVEV is less favourable, with decades between outbreaks of human disease or detection in mosquitoes or sentinel chicken flocks. Further, enhanced surveillance (including more councils participating in surveillance activities and an increase in the overall number of mosquito traps) over many years, has failed to detect MVEV on a more regular basis. The number of LGAs participating in mosquito surveillance has doubled in recent years, however, still fails to detect MVEV on a more regular occurrence. Many of the detections of MVEV from the current study demonstrate that mosquito populations carrying MVEV were in close proximity to residential areas including the rural cities of Wodonga, Rutherglen, Shepparton, Bendigo, Horsham, Robinvale and Mildura, as well as other smaller towns. If MVEV was circulating in cryptic habitats near these residential areas, spill-over into human populations would be expected on a more frequent basis. This is not the case, with almost 50 years since the last outbreak of MVEV in human populations in Victoria and 12 years since detection is surveillance monitoring programs. Additionally, the theory that localised cryptic circulation of MVEV does not provide a clear mechanism for the movement of the G1A sublineage from northwestern Australia into Victorian cryptic habitats and subsequent spread across Victoria without a migratory host being involved.

The explosive nature of MVEV and emergence over many localities, sometimes hundreds of kilometres apart, more likely indicates an influx of disease moving throughout the landscape, aligning with the hypothesis that migratory waterbirds act as the introductory agent for MVEV across the state. It seems unlikely that cryptic pockets of MVEV have not been detected over the last five decades, yet all detections of MVEV in the latest season occurred within the first 13 weeks of 2023 and occurred simultaneously across regionally isolated LGAs from the Northwest to central regions of Victoria, before expanding right along the Murray River system. Further, the detection of MVEV in Horsham, is interesting due to its isolated location and lack of connectivity to river systems that could potentially link to other areas with viral detections.

There have been instances of localised flooding events in regional Victoria that have not been associated with MVEV outbreaks. If isolated cryptic reservoirs of MVEV exist in Victoria, it would follow that regionally specific outbreaks of MVEV would have occurred in response to localised flooding events leading to enhanced mosquito breeding, amplification of virus in those waterbird populations and transmission to localised populations.

Further, population expansion, increased accessibility to remote localities and land use development do not appear to have led to an increase in detections or human cases of flavivirus infections in the southeast of Australia, although sub-clinical cases may not have been tested or reported. The expansion of the VADCP mosquito surveillance program into additional LGAs over the past decade also has not led to an increase in detection of MVEV on a more regular basis. If MVEV is circulating annually in small cryptic habitats, it may well be that these locations have not or are not being sampled as part of the surveillance system.

In northern Australia, vertical transmission of MVEV has been documented in *Aedes tremulus* eggs [17] suggesting a pathway for the “overwintering” of MVEV from one mosquito season to the next. This is scientifically possible with many of the Victorian *Aedes* mosquitoes producing desiccant resistant eggs that hatch in multiple cohorts associated with regular flooding or tides. However, there have been limited studies on survival periods for Australian desiccant resistant mosquito eggs and the role of vertical transmission in MVEV emergence [48]. This theory is unlikely in south-eastern Australia due to the significant periods observed between outbreaks or detections of MVEV. To date there have not been consecutive years of MVEV detected in Victoria, indicating that the role of vertical transmission is unlikely to play a major role in the maintenance nor re-emergence of MVEV between seasons in a Victorian context.

The environmental conditions experienced during October 2022 including a third consecutive La Niña weather system, a positive Southern Oscillation Index, and significant rainfall across multiple LGAs led to vast areas within the Murray River becoming flooded for many months. Mosquito populations increased in response to flooding, initially led by the bird biting *Cx. australicus* and followed in the warmest months by the known MVEV vector *Cx. annulirostris.* Furthermore, linkage of the mosquitoes to the G1A previously only reported from the northwest of Australia suggests that the re-emergence of MVEV in Victoria occurred through migratory water birds, responding to the rapid increase in freshwater resources across south-eastern Australia, allowing the reintroduction of the virus into many geographically separated areas at approximately the same time. In conjunction, the development of the “perfect swarm” of mosquito species, initiated with the development of *Cx. australicus* for the following two months after flooding and then the dominance of *Cx. annulirostris* enabled amplification and a transmission pathway to develop that impacted public health. Future risk assessments should review flooding events associated with positive phases of the Southern Oscillation Index during spring and enhancement of *Cx. australicus* and *Cx. annulirostris* populations as indicators of potential MVEV outbreaks.

## Supporting information

S1 Table List of positive MVEV detections from mosquito pools across Victoria including mosquito collection date, epidemiological week of the year (starting 1 January 2023); Local Government Area from which mosquitoes were collected, unique site number for each trap, method of sample preparation, pool size and Ct score of positive traps. (DOCX)
