# Supplementary material for: “The Perfect Swarm” – Flooding across Northern Victoria leads to intensified mosquito breeding and subsequent re-emergence and transmission of Murray Valley encephalitis virus during the 2022-23 mosquito season
Source: PLoS Negl Trop Dis. 2025 Aug 18;19(8):e0013407. doi: 10.1371/journal.pntd.0013407 (PMC12370201; doi:10.1371/journal.pntd.0013407)
Supplement: S1 Table — (DOCX) [file pntd.0013407.s001.docx]

**Supporting Information**

**S1 Table:** List of positive MVEV detections from mosquito pools across Victoria including mosquito collection date, epidemiological week of the year (starting 1 January 2023); Local Government Area from which mosquitoes were collected, unique site number for each trap, method of sample preparation, pool size and Ct score of positive traps.

| Mosquito collection date | Epi Week | Local Government Area | Site Number | Sample preparation method | Pool size (number of mosquitoes) | Ct |
| --- | --- | --- | --- | --- | --- | --- |
| 4/01/2023 | 1 | Mildura Rural City Council | MIL-8 | Whole trap grinds | 1,000 | 22.29 |
| 5/01/2023 | 1 | City of Greater Bendigo | BEN-1 | Whole trap grinds | 86 | 22.67 |
| 10/01/2023 | 2 | Indigo Shire Council | IND-4 | Whole trap grinds | 933 | 30.6 |
| 10/01/2023 | 2 | Loddon Shire Council | LOD-3 | Whole trap grinds | 411 | 24.98 |
| 10/01/2023 | 2 | Mildura Rural City Council | MIL-9 | Whole trap grinds | 1,000 | 29.39 |
| 10/01/2023 | 2 | Mildura Rural City Council | MIL-10 | Whole trap grinds | 572 | 29.38 |
| 17/01/2023 | 3 | Campaspe Shire Council | CAM-2 | Whole trap grinds | 24 | 36.15 |
| 17/01/2023 | 3 | Loddon Shire Council | LOD-1 | Whole trap grinds | 1,000 | 29.42 |
| 17/01/2023 | 3 | Loddon Shire Council | LOD-2 | Whole trap grinds | 1,000 | 30.67 |
| 17/01/2023 | 3 | Mildura Rural City Council | MIL-1 | *Cx. annulirostris* only | *147* | *23.12* |
| 17/01/2023 | 3 | Mildura Rural City Council | MIL-5 | *Cx. annulirostris* only | *150* | *23.72* |
| 17/01/2023 | 3 | Mildura Rural City Council | MIL-5 | Whole trap grinds | 347 | 25.66 |
| 17/01/2023 | 3 | Mildura Rural City Council | MIL-6 | *Cx. annulirostris* only | *275* | *25.12* |
| 17/01/2023 | 3 | Mildura Rural City Council | MIL-7 | *Cx. annulirostris* only | *150* | *26.92* |
| 17/01/2023 | 3 | Mildura Rural City Council | MIL-8 | Whole trap grinds | 1,000 | 26.77 |
| 17/01/2023 | 3 | Mildura Rural City Council | MIL-8-Pool 2 | Whole trap grinds | 934 | 31.73 |
| 17/01/2023 | 3 | Mildura Rural City Council | MIL-10 | Whole trap grinds | 1,000 | 26.97 |
| 17/01/2023 | 3 | Mildura Rural City Council | MIL-10-Pool 2 | Whole trap grinds | 224 | 21.5 |
| 17/01/2023 | 3 | Greater Shepparton City Council | SHE-2 | Whole trap grinds | 1,000 | 30.4 |
| 18/01/2023 | 3 | Indigo Shire Council | IND-3 | Whole trap grinds | 1,000 | 26.05 |
| 18/01/2023 | 3 | Indigo Shire Council | IND-3-Pool 2 | Whole trap grinds | 1,000 | 27.22 |
| 18/01/2023 | 3 | Indigo Shire Council | IND-5 | Whole trap grinds | 1,000 | 24.53 |
| 19/01/2023 | 3 | Indigo Shire Council | IND-1 | Whole trap grinds | 108 | 33.13 |
| 24/01/2023 | 4 | Campaspe Shire Council | CAM-1 | Whole trap grinds | 1,000 | 34.83 |
| 24/01/2023 | 4 | Indigo Shire Council | IND-2 | Whole trap grinds | 814 | 27.41 |
| 24/01/2023 | 4 | Loddon Shire Council | LOD-1 | Whole trap grinds | 889 | 34.06 |
| 24/01/2023 | 4 | Mildura Rural City Council | MIL-2 | Whole trap grinds | 351 | 23.88 |
| 24/01/2023 | 4 | Mildura Rural City Council | MIL-5 | Whole trap grinds | 374 | 22.89 |
| 24/01/2023 | 4 | Mildura Rural City Council | MIL-10 | Whole trap grinds | 259 | 26.26 |
| 24/01/2023 | 4 | Greater Shepparton City Council | SHE-1 | Whole trap grinds | 815 | 36.65 |
| 24/01/2023 | 4 | City of Wodonga | WOD-1 | Whole trap grinds | 34 | 34.22 |
| 24/01/2023 | 4 | Horsham Rural City Council | HOR-1 | Whole trap grinds | 66 | 20.23 |
| 25/01/2023 | 4 | Indigo Shire Council | IND-5 | *Cx. annulirostris* only | *906* | *32.14* |
| 31/01/2023 | 5 | Campaspe Shire Council | CAM-2 | *Cx. annulirostris* only | *50* | *20.08* |
| 31/01/2023 | 5 | Mildura Rural City Council | MIL-7 | *Cx. annulirostris* only | *128* | *32.92* |
| 31/01/2023 | 5 | Mildura Rural City Council | MIL-8 | *Cx. annulirostris* only | *150* | *20.54* |
| 31/01/2023 | 5 | Mildura Rural City Council | MIL-8 | Whole trap grinds | 350 | 32.9 |
| 31/01/2023 | 5 | Mildura Rural City Council | MIL-10 | *Cx. annulirostris* only | *141* | *22.8* |
| 1/02/2023 | 5 | Loddon Shire Council | LOD-1 | Whole trap grinds | 368 | 23.87 |
| 1/02/2023 | 5 | Loddon Shire Council | LOD-2 | Whole trap grinds | 168 | 21.89 |
| 7/02/2023 | 6 | Mildura Rural City Council | MIL-8 | Whole trap grinds | 328 | 22.59 |
| 7/02/2023 | 6 | Mildura Rural City Council | MIL-10 | Whole trap grinds | 356 | 23.59 |
| 7/02/2023 | 6 | Greater Shepparton City Council | SHE-2 | Whole trap grinds | 9 | 23.05 |
| 7/02/2023 | 6 | Swan Hill Rural City Council | SWA-1 | Whole trap grinds | 94 | 22.95 |
| 14/02/2023 | 7 | Mildura Rural City Council | MIL-10 | *Cx. annulirostris* only | *150* | *20.65* |
| 15/02/2023 | 7 | Indigo Shire Council | IND-6 | Whole trap grinds | 246 | 25.65 |
| 21/02/2023 | 8 | Mildura Rural City Council | MIL-3 | *Cx. annulirostris* only | *16* | *19.2* |
| 21/02/2023 | 8 | Mildura Rural City Council | MIL-4 | *Cx. annulirostri*s only | *166* | *25.32* |
| 21/02/2023 | 8 | Mildura Rural City Council | MIL-10 | Whole trap grinds | 349 | 25.31 |
| 7/03/2023 | 10 | Mildura Rural City Council | MIL-8 | *Cx. annulirostris* only | *76* | *26.02* |
| 7/03/2023 | 10 | Mildura Rural City Council | MIL-10 | *Cx. australicus* only | *19* | *25.26* |
| 15/03/2023 | 11 | Rural City of Wangaratta | WAN-1 | Whole trap grinds | 13 | 25.88 |
| 28/03/2023 | 13 | Gannawarra Shire Council | GAN-1 | Whole trap grinds | 5 | 36.7 |
